# Role of Thrombin in Central Nervous System Injury and Disease

**DOI:** 10.3390/biom11040562

**Published:** 2021-04-12

**Authors:** Nathan A. Shlobin, Meirav Har-Even, Ze’ev Itsekson-Hayosh, Sagi Harnof, Chaim G. Pick

**Affiliations:** 1 Department of Neurological Surgery, Feinberg School of Medicine, Northwestern University, Chicago, IL 60611, USA; 2 Department of Anatomy and Anthropology, Sackler Faculty of Medicine, Tel Aviv University, Tel Aviv 6997801, Israel; 3 Sylvan Adams Sports Institute, Tel Aviv University, Tel Aviv 6997801, Israel; 4 Department of Physiology and Pharmacology, Sackler Faculty of Medicine, Tel Aviv University, Tel Aviv 6997801, Israel; Zeev.ItsekzonHayosh@sheba.health.gov.il; 5 Department of Neurology and Joseph Sagol Neuroscience Center, The Chaim Sheba Medical Center, Tel HaShomer 5262000, Israel; 6 Department of Neurosurgery, Beilinson Hospital, Rabin Medical Center, Tel Aviv University, Petah Tikva 4941492, Israel; sagiha@clalit.org.il; 7 Sagol School of Neuroscience, Tel Aviv University, Tel Aviv 6997801, Israel; 8 Center for Biology of Addictive Diseases, Tel Aviv University, Tel Aviv 6997801, Israel

**Keywords:** blood–brain barrier, glioblastoma, neurodegenerative diseases, neuroinflammation, protease-activated receptor, prothrombin, TBI, thrombin

## Abstract

Thrombin is a Na^+^-activated allosteric serine protease of the chymotrypsin family involved in coagulation, inflammation, cell protection, and apoptosis. Increasingly, the role of thrombin in the brain has been explored. Low concentrations of thrombin are neuroprotective, while high concentrations exert pathological effects. However, greater attention regarding the involvement of thrombin in normal and pathological processes in the central nervous system is warranted. In this review, we explore the mechanisms of thrombin action, localization, and functions in the central nervous system and describe the involvement of thrombin in stroke and intracerebral hemorrhage, neurodegenerative diseases, epilepsy, traumatic brain injury, and primary central nervous system tumors. We aim to comprehensively characterize the role of thrombin in neurological disease and injury.

## 1. Introduction

**Figure 1:**
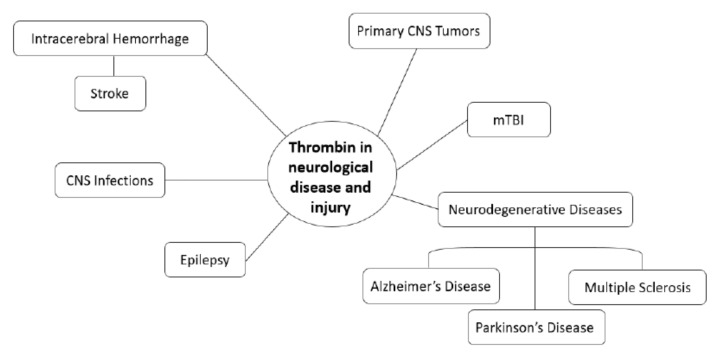
Relevance of thrombin to neurological disease and injury. The role of thrombin in epilepsy, CNS infections, mild traumatic brain injury, neurodegenerative diseases, stroke, and primary CNS tumors continues to be investigated.

Thrombin is a Na^+^-activated allosteric serine protease of the chymotrypsin family central to blood coagulation with further involvement in inflammatory responses, cell protection, and apoptosis [1,2,3,4,5,6]. Thrombin is a large, spherical molecule, with a groove, consisting of a light A-chain and heavy B-chain with two anion-binding exosites [7,8,9]. The structure of thrombin allows binding of substrates to promote signaling through a myriad of different pathways [7,10,11]. Exosite I recognizes fibrinogen, fibrin, factor Va, thrombomodulin, and hirudin, while exosite II recognizes glycosaminoglycans, including heparin sulfate and glycoprotein Ibα [8,9]. Prothrombin, the inactive precursor of thrombin, is generated within the liver for circulation in the plasma until conversion into active thrombin in the coagulation cascade [8,12]. The serine protease inhibitor antithrombin III is responsible for irreversible inactivation of thrombin via interaction with heparan sulfates, while exogenous compounds including hirudin and heparin may inhibit thrombin by binding to exosites [8,13,14,15,16]. Thrombin is involved in wound healing, arterial and venous thrombosis, and the pathogenesis of conditions, including atherosclerosis, sepsis, and cancer [17]. It is clear that thrombin is involved in neurological disease [8,18]. In this review, we explore the mechanisms of thrombin action, localization, and functions in the central nervous system (CNS) and describe the involvement of thrombin in stroke and intracerebral hemorrhage, neurodegenerative diseases, epilepsy, traumatic brain injury, and primary CNS tumors. We aim to comprehensively characterize the role of thrombin in neurological disease and injury (Figure 1).

## 2. Mechanisms of Thrombin Action

### 2.1. Coagulation

In the coagulation cascade, tissue factor complexes with factor VIIIa, generating factors IXa and Xa [1,2,3,17,19]. Small quantities of Xa generate miniscule quantities of thrombin, activating factor XI, and cofactors VIII and V [1,2,3,17,19]. The VIII–IXa complex generates sufficient Xa to promote the formation of the prothrombinase complex, consisting of factors Va and Xa, Ca^2+^, and anionic phospholipids [1,2,3,17,19]. Within the prothrombinase complex, factor Xa proteolytically cleaves prothrombin to meizothrombin, with further cleavage to thrombin [1,2,3,17,19]. Paradoxically, thrombin exerts both procoagulant and anticoagulant effects [1,2,3,17,19]. In the procoagulant pathway, thrombin converts fibrinogen to an insoluble fibrin clot to which platelets adhere in order to begin wound repair [1,2,3,17,19]. Activation of transglutaminase factor XIII to stabilize the fibrin clot, TAF1 inhibition of fibrinolysis, and proteolytic action of factors V, VIII, and XI reinforce the procoagulant actions [1]. In the anticoagulant pathway, thrombin interacts with protease-activated receptors (PAR), a family of G-protein coupled receptors activated through cleavage of a portion of an extracellular domain. Binding of thrombin to thrombomodulin augments the activation of protein C on the endothelial protein C receptor (ECPR) while restricting cleavage of fibrinogen and PAR1 [1,2,3,17,19]. Protein C cleaves and inactivates factors Va and VIII to prevent further thrombin generation, while the serine protease inhibitor antithrombin irreversibly inhibits thrombin along with heparin [1,2,3,17,19]. These divergent actions promote a balance between the procoagulant and anticoagulant pathways [1,2,3,17,19].

### 2.2. Thrombotic and Immune Functions

Pleiotropic effects of thrombin have been described primarily in in vitro studies [17]. Thrombin affects the activity of platelets, fibroblasts, vascular smooth muscle cells, endothelial cells, monocytes, and T lymphocytes [20,21,22,23,24]. Thrombin exerts mitogenic activity on vascular smooth muscle cells and endothelial cells via PARs and modulates vascular permeability, vascular tone, and angiogenesis [17,25,26,27,28,29,30,31,32,33]. Mitogenic effects result from the action of basic fibroblast growth factor and sequestering of thrombin in subendothelial basement membranes [29,30]. Changes in vascular tone are secondary to PAR1-initiated endothelial-dependent relaxation mediated by nitric oxide and polarizing factors [33]. Increases in vascular permeability occur due to changes in the distribution of vascular endothelial-cadherin and associated catenins and actin–myosin interactions [31,32]. This results in plasma protein leakage, creating a proangiogenic matrix [34,35,36]. Decreased cyclic adenosine monophosphate-mediated attachment of endothelial cells promotes angiogenesis coupled with potentiation of vascular endothelial growth factor (VEGF)-induced endothelial cell regulation, increased transcription of VEGF via the production of reactive oxygen species (ROS) and expression of hypoxia-inducible factor 1, upregulation of VEGF receptor expression, and increased levels of αV/β3 integrin [37,38,39,40,41]. Thrombin promotes inflammation and intimal hyperplasia via dysfunctional mixed phenotype intermediates of vascular progenitors and the activation of monocytes, T lymphocytes, mast cells, and endothelial cells [42,43]. Thrombin also affects processes involved in tissue repair, including PAR-1 mediated induction of cytokines promoting angiogenesis, leukocyte migration, and edema formation [34,44,45].

### 2.3. Cellular Protection and Apoptosis

**Figure 2:**
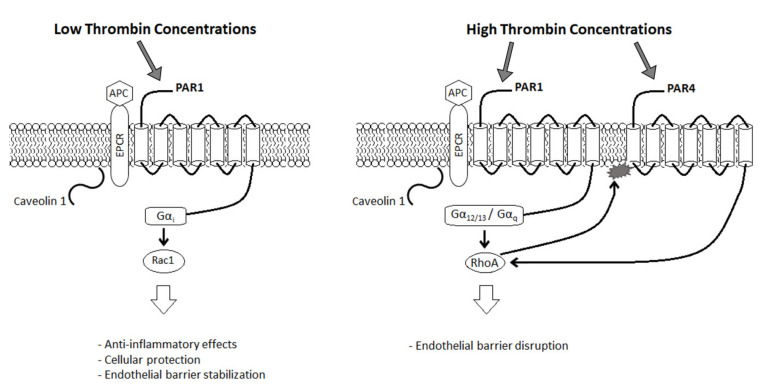
Role of thrombin in cellular protection and apoptosis. At low concentrations, thrombin has anti-inflammatory effects and is involved in cellular protection and endothelial barrier stabilization. At high concentrations, thrombin leads to endothelial barrier disruption.

Thrombin is involved in cell protection and induces apoptosis through the activation of PAR1 via the guanosine triphosphate binding protein RhoA and the protein kinase C pathway [11,46,47]. PAR1 is activated via cleavage by thrombin into tethered activation peptide ligands that enable transmembrane signaling [48]. After cleavage, some PAR1 receptors are internalized, while approximately 20–60% remain on the cell surface [49]. The activity of thrombin depends on thrombin concentration and length of exposure of thrombin to the cellular environment (Figure 2). PAR1 receptors are cleaved more rapidly at high thrombin concentrations, enabling a greater cellular response [49]. Short exposure to low thrombin concentrations activates pathways for cellular protection, while prolonged exposure to thrombin stimulates apoptosis [4]. Given the ability of PARs to form homodimers and heterodimers, the specific dimer also determines which signal transduction cascade is active [50,51]. Interaction of the activated protein C (APC) with endothelial protein C receptor (EPCR) promotes anti-inflammatory effects, cellular protection, and endothelial barrier stabilization by switching PAR1 signal transduction to cytoprotective and regenerative functions [46,52,53,54]. EPCR is also involved in endocytosis via interaction with the lipid raft plasma membrane protein caveolin-1 [4]. Binding of the APC or protein C to EPCR prompts activation of PAR1 after dissociation of EPCR from caveolin 1 [4]. At low thrombin concentrations, PAR1 activation is mediated by the APC–EPCR, promoting cytoprotective Gα_i_ signaling and subsequent Rac1 activation. High thrombin concentrations promote PAR1 activation via Gα_q_ and Gα_12/13_ signaling and subsequent RhoA activation and eliminate cytoprotective effects of the APC-EPCR via activation of PAR4 [4,46,52,54,55,56]. The combined effect is a switch to thrombin-mediated cellular degeneration [46]. Additionally, scaffold proteins β-arrestin 1 and 2 are involved in signaling dependent on and independent of G-protein-coupled receptors [4]. A dose-dependent switch mediates the transition to G-protein-independent signaling, inducing cytoskeletal organization [57,58,59]. However, activation of cellular pathways via a receptor depends on ligand bias, receptor bias, and cell bias, indicative of specific conditions that promote the activation of distinct pathways that have yet to be determined [4,60].

## 3. Thrombin in the Central Nervous System

### 3.1. Localization in Brain

Prothrombin and thrombin have been localized to neurons and glial cells in the CNS [61]. Similarly, factor X and inhibitors of thrombin activity, including antithrombin III and the protease nexin-1 (PN-1), are locally expressed in the brain, with much of the PN-1 localized around blood vessels [62,63,64,65]. Prothrombin mRNA expression is greatest in the cerebral cortex and moderate in the hippocampus and cerebellum [66]. The hippocampus displays greater labeling of thrombin in the pyramidal layers and lower labeling in fiber layers on immunohistochemistry [67]. Prothrombin expression and thrombin activity in the nervous system are highly regulated in physiological and pathological states [68,69]. Similarly, PARs are expressed in neurons, oligodendrocytes, microglia, and astrocytes [61,70,71,72], indicating that thrombin may be active in these cells under physiological conditions. Similarly, PAR1 is found in the pyramidal layers of the hippocampus, and PAR3 and PAR4 are found in all cortical layers and the thalamus of rat brains [71]. Rat neurons and astrocytes contain all PAR receptors [73,74]. While thrombin utilizes all four PARs to engage signal transduction pathways, PAR1 activation is primarily responsible for the actions of thrombin in the CNS [8]. High concentrations of thrombin increase brain damage, while low thrombin concentrations and thrombin preconditioning are neuroprotective [61,75].

### 3.2. Neuro-Physiological Functions

Thrombin has myriad physiological functions in the CNS. First, thrombin plays a role in neuronal development, given the colocalization and similar developmental pattern of prothrombin mRNA and PAR1 in the rat brain [76,77]. Second, thrombin exerts mitogenic functions to enable cellular proliferation and differentiation—as described above. Mitogen-activated protein kinases (MAPKs) regulate cellular proliferation and differentiation, while PAR1 mediates the mitogenic action in astrocytes and microglia [73,78,79,80]. Nerve growth factor and endothelin-1 synthesis enable astrocyte proliferation [81,82,83]. Additional signal transduction cascades specific to cell type involve the PI 3-kinase pathway and phospholipase C (PLC)/Ca2+/protein kinase C (PKC) pathway [84]. Third, thrombin signaling alters cell morphology and enables cell migration. Thrombin alters the morphology of astrocytes, fetal neurons, and neuroblastoma cells via neurite outgrowth, stellate retraction, and cytoskeleton rearrangement to enable growth cone guidance and cell migration [81,82,83,85,86]. Fourth, thrombin regulates synaptic transmission. This has been demonstrated particularly in the CA3 layer of the hippocampus but not the CA1 layer [87]. Fifth, thrombin regulates synaptic plasticity [87]. Thrombin affects long term potentiation in the hippocampus through a series of pathways mediated by PAR1 [88,89,90,91,92,93,94,95]. High thrombin concentrations prompt a slow onset long-term potentiation dependent on the N-methyl-D-aspartate (NMDA) receptor [88,89]. Low thrombin concentrations promote long-term potentiation dependent on voltage-gated calcium channels and mGluR-5 through APC [88,89].

### 3.3. Neuro-Pathological Functions

Thrombin also plays a role in pathological processes in the CNS. Thrombin action is greatest under conditions leading to disruption of the blood–brain barrier (BBB) [96]. Thrombin induces damage to the BBB and an associated increase in permeability through a variety of mechanisms. Thrombin leads to F-actin fiber increase, destruction of tight junction, nitric oxide release, and PAR-1 dependent production of mitochondrial and cytosolic ROS [97]. Disruption of the BBB also results from thrombin-related expression of matrix metalloproteinases (MMPs) in brain tissue, particularly in pericytes and in activated microglia [98,99,100]. Similarly, thrombin is involved in brain inflammation. Thrombin activates microglia through p38 MAPK and c-Jun N-terminal kinase (JNK) in processes mediated by PAR1, PAR3, and PAR4 and has been associated with glial scar formation [73,74,79,101]. Thrombin also activates astrocytes through pro-inflammatory compounds, including arachidonic acid, nitric oxide, the chemokine growth-regulated oncogene/cytokine-induced neutrophil chemoattractant-1, and interleukin (IL)-8 [102,103]. Thrombin increase enhances the expression and release of pro-inflammatory factors, such as CD-40, cyclooxygenase 2, inducible nitric oxide synthase, tumor necrosis factor-α, IL-1α/β, IL-6, and IL-12 [79]. These cytokines may exert positive feedback on thrombin production [104]. Additionally, thrombin promotes neurotoxicity. Thrombin induces shrinkage of striatal tissue dependent on activation of microglia, extracellular signal-related kinase, and MAPK-related pathways [105,106]. Concomitant exposure to MMP-9 increases neurotoxicity as PAR1 converts pro-MMP-9 to active MMP-9 [107,108]. Thrombin also cleaves apolipoprotein E (apoE) to a fragment with a cytotoxic domain [109,110]. Conversely, injured brain cells release neurotoxic thrombin [111]. Moreover, thrombin promotes maladaptive synaptic plasticity that may result in seizures [89,112]. Furthermore, thrombin is involved in intracerebral coagulation, as in the remainder of the body [113]. These pathological functions, mediated by thrombin in neurological conditions and injuries, will be further elaborated.

## 4. Neurodegenerative Diseases

### 4.1. Alzheimer’s Disease

The role of thrombin in Alzheimer’s disease (AD) has been well characterized. Thrombin accumulates in senile plaques, amyloid deposits, neurofibrillary tangles, and microvessels in the brains of AD patients, while prothrombin mRNA is expressed in neurons and glial cells [114,115]. In a rat model, the modulation of PAR (particularly PAR1) expression was demonstrated in hippocampal astrocytes and microglia [116]. AD also promotes the release of thrombin through β-amyloid (Aβ) activation of factor XII and release of the transcription factor hypoxia inducible factor 1α (HIF-1α) [117,118,119]. Thrombin represents a convergence point for AD risk factors [120]. In vitro experiments have demonstrated intracellular aggregates of the microtubule-associated tau protein in hippocampal neurons arising from the ability of thrombin to proteolyze tau protein but inability to process phosphorylated tau protein [121]. Neurotoxic tau deposits leading to apoptosis of hippocampal neurons result from thrombin-mediated hyperphosphorylation and accumulation of tau via the of PAR1/4 and the extracellular signal-regulated kinase (ERK)1/2 pathway [121,122]. Thrombin also cleaves β-amyloid precursor protein, promoting Aβ accumulation [123,124]. Intracellular Ca^2+^ influx and oxidative stress compound Aβ neurotoxicity [125]. Although PN-1 protects neurons against neurotoxicity resulting from Aβ accumulation, thrombin–PN-1 complex formation attenuates the activity of PN-1 [126,127]. Thrombin promotes microglial NAPDH oxidase production of ROS and expression of pro-inflammatory IL-8 and integrins, two key pathophysiological processes in AD, leading to further cell death [8,118,128]. Intracerebral administration of thrombin in rats increases apoE levels, leading to Aβ accumulation and ensuing cognitive difficulties [119]. The direct thrombin inhibitor dabigatran reduces expression of inflammatory cells, markers of oxidative stress including ROS, and tau pathology in vivo [129,130]. Accordingly, investigators have suggested the use of direct thrombin inhibitors in the treatment of AD due to their selectivity in inhibiting thrombin and relatively mild side effect profile [131]. However, whether thrombin prompts neurodegeneration and leads to formation of amyloid plaques and neurofibrillary tangles in vivo remains to be seen.

### 4.2. Parkinson’s Disease

Thrombin is associated with modulation of neurological injury and progression of Parkinson’s disease (PD). Prothrombin and PAR1 are upregulated in astrocytes positive for glial fibrillary acid protein in the brains of PD patients, and expression of PARs is increased in the vessel wall of the substantia nigra pars compacta [8]. Loss of dopaminergic cells, neuroinflammation, and oxidative stress occur after thrombin injection into the substantia nigra [132,133,134,135,136]. Expression of pro-apoptotic proteins caspase-3 and p53 is upregulated in dopaminergic neurons of the substantia nigra, while pro-inflammatory molecules including nitric oxide, IL-1α/β, IL-6 and TNF-α are expressed due to microglial activation by thrombin [8,132,134,136]. The timing and dose of thrombin administration is important in understanding the pathophysiological role of thrombin in PD. Delayed preconditioning with thrombin protects against cell damage resulting from infusion of 6-hydroxydopamine [137]. Coadministration of 6-hydroxydopamine and thrombin or PAR1 agonists amplifies neuronal damage and behavioral deficits, while previous treatment with PAR1 antagonists eliminates the neuroprotective effects of preconditioning [137]. However, thrombin neurotoxicity may also be independent of the PAR1 pathway, and PAR1 activation may be neuroprotective given the absence of PAR1 in microglia in the substantia nigra pars compacta [8,138]. Upregulation of PAR1 in astrocytes may have restorative action on dopaminergic neurons, preventing their degeneration and cell death in PD [139]. Similarly, thrombin preconditioning reduces dopaminergic terminal loss and ventricular enlargement, while PAR1 antagonists exacerbate neurological deficits resulting from 6-hydroxydopamine administration [140,141]. Interestingly, in the substantia nigra pars compacta of patients with PD, neurons, microglia, and oligodendrocytes lack PARs [139]. However, PAR4 is involved in dopaminergic neuron loss in the substantia nigra upon striatal thrombin injection [71], indicating PAR4 may modulate thrombin-related pathogenesis in PD. As with AD, dabigatran, a selective thrombin inhibitor widely prescribed as an oral anticoagulant, has been utilized in animal models of PD. Dabigatran suppresses thrombin accumulation in the substantia nigra, decreasing the expression of pro-inflammatory cytokines and reducing oxidative stress [142,143]. Lipopolysaccharide-binding protein has also been shown to reverse the amyloid accumulation of fibrin induced by bacterial lipopolysaccharide and occurring in the blood of patients with PD [144,145]. Additional studies are necessary to characterize the utility of these treatments.

### 4.3. Multiple Sclerosis

The role of thrombin in multiple sclerosis (MS) is less clear than in AD or PD. Progressive axonal loss in animal models of MS is associated with fibrin accumulation in cerebral vasculature [146]. Thrombin activity is associated with BBB disruption, microglial activation, inflammatory demyelination, and axonal damage, indicating that thrombin may be involved in the pathogenesis of MS [147]. Mouse models of experimental autoimmune encephalitis (EAE), the most commonly used experimental model for MS, demonstrate a large increase in thrombin activity prior to appearance of motor impairment [147,148,149]. Thrombin activity continues to increase until the peak of clinical disease [147,148,149]. Thrombin activity begins in the early stage of the disease preceding demyelination and is associated with the progression of disease [147]. Thrombin activity and fibrin deposition are closely associated with increased microglial activation and the extent of demyelination [147]. Studies examining human patients have not reported increased thrombin in the CNS [150]. However, PAR1 mRNA expression and surface density in platelets and megakaryocytes and plasma factor XII activity are greater in patients with MS [151,152,153]. Similarly, oligodendrocytes express PAR1 receptors, while PAR2 activation secondary to the action of macrophages promotes oligodendrocyte death in the EAE model [154,155]. Additionally, EAE animals exhibit increased BBB permeability early in the clinical course [156]. Given that PAR1 inhibitors preserve the BBB and reduce demyelination and inflammatory infiltration of the CNS while decreasing thrombin levels in an EAE mouse model, PAR1 inhibitors may play a role in treatment of MS [157]. Early PN-1 changes may also be a target for thrombin-modulating drugs in MS [149]. Temporal pharmacological enhancement of endogenous APC generation via a selective recombinant protein C activator thrombin analog may represent another treatment option [158]. However, future studies are necessary to completely characterize the role of thrombin in the neuroinflammation present in MS.

## 5. Intracerebral Hemorrhage and Stroke

Thrombin is involved in stroke and intracranial hemorrhage (ICH). Elevated thrombin increases the risk of acute ischemic stroke [159]. Conversely, ischemia leads to increased levels of thrombin and prothrombin via activation of factor Xa, and increased BBB permeability permits thrombin influx from the bloodstream [8,160,161]. Thrombin activity is associated with infarct volume [162]. Similarly, thrombin activity eluted from clots originating from patients with atrial fibrillation differs from that of patients with atherosclerosis, indicating its potential role as a diagnostic marker [163]. Thrombin exerts dose-dependent effects. Low concentrations protect neurons and astrocytes in the hippocampus against oxygen glucose deprivation, hypoglycemia, and ROS, while higher concentrations promote hippocampal and motor neuron cell death due to unknown mechanisms of PAR1 neurotoxicity [8,164,165,166]. Focal ischemia and ICH can cause intracerebral edema associated with an increase in thrombin [113,167,168,169]. Thrombin determines the release of pro-inflammatory factors, including iron and MMPs [79,136,168,170]. Iron increases the risk of brain edema after ICH, as transferrin containing iron overwhelms neuroprotective effects at low thrombin concentrations [171,172]. MMPs augment damage to neural tissue [107]. Separately, expression of pro-apoptotic proteins such as Bim due to increased cyclin-dependent kinase 4 stimulation in hypoxic conditions facilitates apoptosis [173,174]. PAR1 is primarily responsible for mediation of thrombin activity, despite complex regulation of PAR1, PAR3, and PAR4 [8]. PAR1 activation promotes disruption of the extracellular matrix and increased permeability of the BBB in addition to neuronal damage and increased size of infarcts [72,175]. NMDA receptor stimulation via increased astrocyte glutamate release secondary to PAR1 activation may also lead to excitotoxicity and subsequent BBB disruption [91,176,177]. The formation of glial scars via PAR1 activation after stroke or ICH decreases the potential for regeneration of neural tissue [101,178]. Thrombin preconditioning reduces the size of the infarct in ischemic stroke and edema in ICH [68,179,180]. PAR1 antagonists eliminate thrombin preconditioning-mediated neuroprotection [75]. The effect of hirudin is equivocal, as authors have described reversal of neuroprotective thrombin preconditioning, while others have described reduced infarct volumes upon administration following occlusion of the middle cerebral artery [181,182]. Thrombin preconditioning may include ceruloplasmin upregulation to promote tolerance to edema, and JNK may promote neuroprotection [183,184]. The balance between thrombin and PN-1 may augment repair processes in stroke, given unchanged expression levels under ischemic conditions [185,186]. Although dabigatran is utilized in stroke prophylaxis in patients with atrial fibrillation, its efficacy relative to other treatments such as rivaroxaban is equivocal [187,188,189]. Determining the optimal dosing of dabigatran and utility with concomitant antiplatelet therapy may be particularly important [190]. Future studies should continue to identify mechanisms of thrombin involvement in stroke and ICH.

## 6. CNS Infections

Thrombin has been implicated in infections with neurological symptomatology. Translocation of the BBB is an important step in the pathogenesis of CNS infections [191,192]. Increased permeability of the BBB due to thrombin increases the ability of pathogens to cross the blood–brain barrier. Meningitis-causing microorganisms enter the brain through transcellular penetration of the BBB [191]. Thrombin-activated fibrinolysis inhibitor (TAFI) likely mediates inhibition of the complement system and activation of systemic complications and inflammation in pneumococcal meningitis [193]. Similarly, TAFI genotype is associated with the risk of meningococcal disease and death [194]. Activation of TAFI mediates disseminated intravascular coagulation and sepsis in patients with meningococcal sepsis [195]. Expression of endothelial thrombomodulin and endothelial protein C receptor is lower in patients with meningococcal sepsis, emphasizing the role of dysfunction of the thrombin-mediated anticoagulation pathways in severe disease [196]. Additionally, the role of thrombin in CNS infections is best characterized in the pathogenesis of human immunodeficiency virus (HIV). Thrombin generation rises by 24–48% while anticoagulants including antithrombin and protein C decrease in computational models of untreated HIV infection [197]. A concentration-dependent mechanism may underlie acceleration of the HIV-induced cell-fusion rate by thrombin [8]. HIV promotes disruption of the BBB, perhaps promoting an influx of prothrombin or thrombin, despite decreased thrombin generation in the plasma [198,199]. HIV also increases coagulation potential, augmenting the risk of ischemic cerebral infarction and intracranial venous thromboembolism and atherosclerotic disease [197]. Thrombin may be involved in the CNS pathogenesis of HIV via stimulation of T cell motility and production of pro-inflammatory cytokines to moderate the cross-talk between the coagulation cascade and adaptive immune system in areas of vascular injury [200]. Recent evidence suggests that thrombin plays a role in the CNS sequelae of HIV. HIV-associated encephalitis and dementia involve neuroinflammation and continual neuronal damage [8]. HIV-dementia involves changes mediated by PARs, including altered morphology, neurite retraction, or neurotoxicity [8]. PAR2 is upregulated in neurons, suggesting involvement of thrombin in HIV-associated dementia [201]. Similarly, patients with HIV-associated encephalitis exhibit increased levels of prothrombin mRNA and protein and upregulated PAR1 in astrocytes [176]. Neuroinflammation in HIV patients may be PAR1-dependent [202]. The direct relationship between thrombin and other neuroinfectious diseases continues to be investigated. Schizont-stage *Plasmodium falciparum* induces low levels of primary human microvascular endothelial cell death and prolongs thrombin-induced barrier disruption [203]. β-arrestin 2, a downstream component of thrombin, protects against neurological dysfunction in herpes simplex virus-1 induced encephalitis [204]. Varicella zoster virus encephalitis may promote a vasculopathy [205]. Additional research is necessary to precisely characterize potential additional mechanisms through which thrombin promotes CNS infections.

## 7. Mild Traumatic Brain Injury (mTBI)

The involvement of thrombin in mild traumatic brain injury (mTBI) has begun to be investigated. Thrombin levels rise until one-hour post-trauma and increase again after 72 h with a concomitant increase in PAR1 at both time points, indicative of astrocyte activation [206]. Similarly, traumatic brain injury is associated with disruption of the BBB, allowing thrombin to enter and promote neurotoxicity [68]. In cases of trauma-induced amnesia, recovery from amnesia occurs when thrombin activity normalizes in the hippocampus [207]. Rescue from trauma-induced amnesia can be accomplished by inhibiting thrombin activity or blocking PAR1 [208]. A recent study determined that downregulation of hippocampal astrocyte glutamate transporters by thrombin following TBI was associated with depression in mice [209]. Inhibition of PAR1 or Rho kinase decreased depressive symptoms [209]. Further investigation regarding the role of thrombin in the pathogenesis of TBI is warranted.

## 8. Epilepsy

The role of thrombin in epilepsy is being increasingly explored. Thrombin increases sensitivity to seizure-like activity [206]. Seizures are commonly associated with conditions that compromise BBB function, including stroke and intracerebral hemorrhage, TBI, and CNS infections through regional destruction of the BBB [210,211,212]. Increased permeability of the BBB stimulates seizures due to increased exposure of the brain to thrombin and other serum components [213]. BBB breakdown activates the coagulation cascade irrespective of the presence of intracerebral hemorrhage, generating additional thrombin [214,215,216]. Uncontrolled seizures, either idiopathic or related to existing brain injury, lead to increased permeability of the BBB and an associated increase in the concentration of thrombin [61]. These factors trigger a positive feedback loop that promotes epilpetogenesis [217]. Stroke may also precipitate the development of post-stroke epilepsy by generating permanent structural changes from which an epileptic focus arises [112]. Additionally, thrombin has direct epileptogenic effects. Thrombin triggers the generation of epileptic seizures by increasing excitatory tone and decreasing inhibition in CA3 neurons of the hippocampus [87]. This process is mediated by PAR1 [89]. Activation may occur in a manner independent of NMDA by amplifying persistent voltage-gated sodium channel current through tetrodotoxin-sensitive channels [218]. Increased thrombin immunofluorescence is visualized in the hippocampus of mice with pilocarpine-induced status epilepticus, while systemic inhibition of thrombin mitigates the behavioral outcome of pilocarpine in this model [219]. Although investigators have suggested that oral anticoagulants that target thrombin and PAR1 may be useful anti-epileptic medications [219], continued research is necessary.

## 9. Primary CNS Tumors

The involvement of thrombin in primary CNS tumors has also been increasingly studied. Generally, thrombin promotes tumor cell adhesion, enhances tumor cell growth, upregulates tumor-related angiogenesis, and increases tumor cell seeding and metastases [38,220,221]. This is also the case in primary CNS tumors [222]. Thrombin activity is increased in high-grade glioma and non-glial malignant CNS tumor cell lines [223]. Meizothrombin stimulates human glioblastoma cells via interaction with PAR1 [224]. Tumor precursor cells derived from primary human gliomas and glioblastoma cells overexpress PAR1 [225,226,227]. PAR-1 is involved in thrombin-induced Ca^2+^ mobilization in human meningioma cells [228]. Similarly, PAR1 inhibition suppresses self-renewal and growth of glioma progenitor cells and gliomas in vivo [225]. PAR1 also mediates protein kinase A (PKA) activation via the nuclear factor kappa-light-chain enhancer of activated B cells (NF-κB)-associated catalytic protein kinase A α subunit rather than cAMP levels in glioblastoma cells [229]. Interestingly, other factors including platelet-derived growth factor may be involved in the pathogenesis of glioblastoma [230]. Thrombin-induced A172 human glioblastoma cell proliferation has been found to depend on the level of platelet-derived growth factor-AB (PDGF-AB) [230]. Anti-PAR1 antibodies do not affect the secretion of PDGF-AB or cell growth [230]. Elevated thrombin activity is associated with brain edema resulting from tumor-induced breakdown of the BBB [223]. These pathomechanisms may lead to the formalization of biomarkers for glioblastoma based on components of the thrombin pathway, such as anti-thrombin [231]. A novel six amino acid chloromethyl-ketone compound that inhibits PAR1 activation decreases glioblastoma proliferation rate, colony formation, and invasion in vitro and increased survival and reduced edema volume formation in rats [232]. Similarly, dabigatran inhibits growth, cell cycle progression, migration, and formation of endothelial tubes in glioblastoma cells [233]. Argatroban reduces glioma mass and prolongs survival [234]. Although additional investigation is required, the thrombin pathway represents a potential target for novel therapeutic strategies in humans.

## 10. Conclusions

Thrombin is involved in coagulation, inflammation, cell protection, and apoptosis. Increasingly, the role of thrombin in the CNS has been explored. Although low concentrations of thrombin are neuroprotective, high concentrations of thrombin exert pathological effects in the CNS through BBB disruption, neuroinflammation, neurotoxicity, maladaptive synaptic plasticity, and coagulation. PAR1 is predominantly responsible for modulating these effects. Involvement of thrombin in neurodegenerative diseases and intracerebral hemorrhage is reasonably well characterized. There is tremendous potential for application of the knowledge regarding thrombin to the management of stroke, particularly with the increased usage of endovascular thrombectomy clot retrieval and the development of novel oral anticoagulants. The roles of thrombin in CNS infections, TBI, epilepsy, and primary CNS tumors continue to be elucidated. Further research will clarify the mechanisms of thrombin pathogenesis in these conditions to catalyze the generation of appropriate therapeutics.
